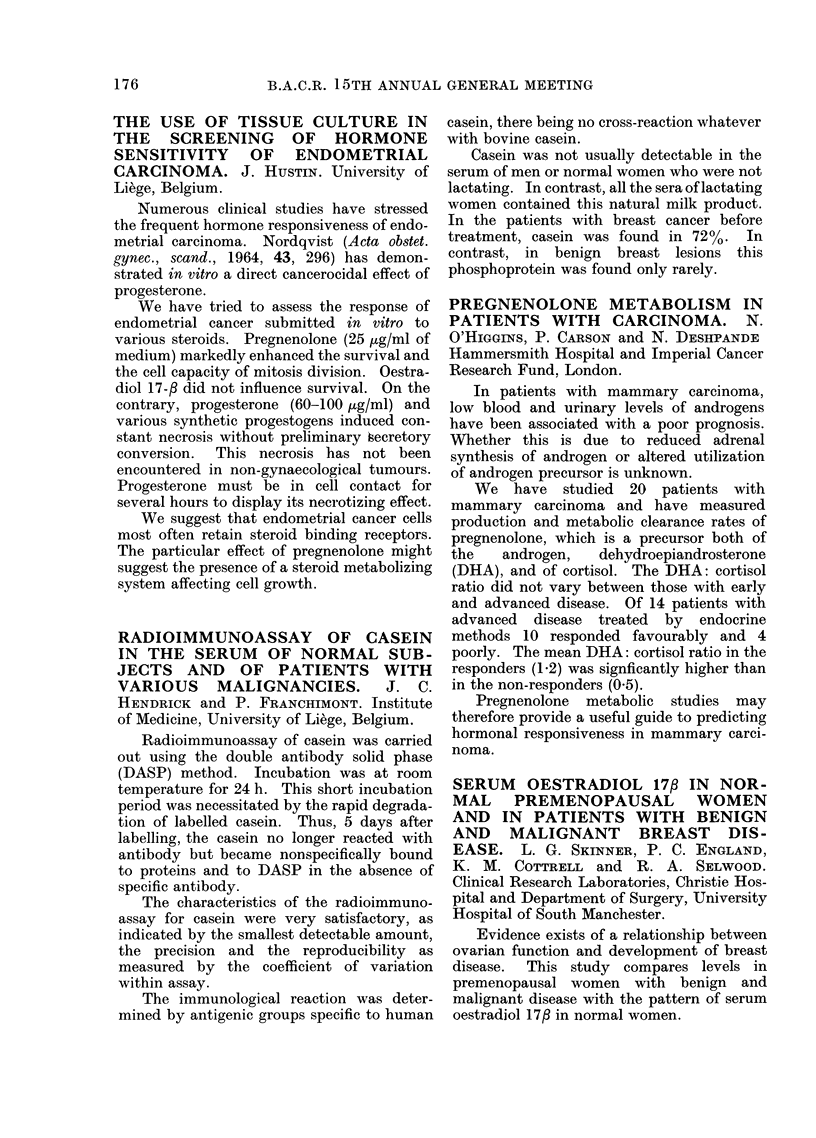# Proceedings: The use of tissue culture in the screening of hormone sensitivity of endometrial carcinoma.

**DOI:** 10.1038/bjc.1974.141

**Published:** 1974-08

**Authors:** J. Hustin


**THE USE OF TISSUE CULTURE IN THE SCREENING OF HORMONE SENSITIVITY OF ENDOMETRIAL CARCINOMA.** J. HUSTIN. University of Liège, Belgium.

Numerous clinical studies have stressed the frequent hormone responsiveness of endometrial carcinoma. Nordqvist (*Acta obstet. gynec., scand.*, 1964, **43**, 296) has demonstrated *in vitro* a direct cancerocidal effect of progesterone.

We have tried to assess the response of endometrial cancer submitted *in vitro* to various steroids. Pregnenolone (25 $\mu$g/ml of medium) markedly enhanced the survival and the cell capacity of mitosis division. Oestradiol 17-$\beta$ did not influence survival. On the contrary, progesterone (60–100 $\mu$g/ml) and various synthetic progestogens induced constant necrosis without preliminary secretory conversion. This necrosis has not been encountered in non-gynaecological tumours. Progesterone must be in cell contact for several hours to display its necrotizing effect.

We suggest that endometrial cancer cells most often retain steroid binding receptors. The particular effect of pregnenolone might suggest the presence of a steroid metabolizing system affecting cell growth.